# Impact of CPAP Therapy Adherence on Time to First Recurrence of Paroxysmal Atrial Fibrillation in Patients with Severe Obstructive Sleep Apnea

**DOI:** 10.3390/life16030389

**Published:** 2026-02-28

**Authors:** Petar Kalaydzhiev, Radostina Ilieva, Natalia Spasova, Slavi Yakov, Dimitar Markov, Neli Georgieva, Elena Kinova, Assen Goudev

**Affiliations:** 1 Department of Emergency Medicine, Medical University of Sofia, 1606 Sofia, Bulgaria; 2 Cardiology Department, University Hospital “Tsaritsa Yoanna—ISUL”, 1527 Sofia, Bulgaria

**Keywords:** atrial fibrillation, obstructive sleep apnea, continuous positive airway pressure, CPAP adherence, paroxysmal atrial fibrillation, arrhythmia recurrence

## Abstract

**Background**: Obstructive sleep apnea (OSA) is a major modifiable risk factor for atrial fibrillation (AF), promoting arrhythmogenesis through intermittent hypoxia, autonomic activation, and atrial remodeling. Although continuous positive airway pressure (CPAP) effectively treats OSA, real-world evidence linking objectively measured CPAP exposure to clinically relevant AF recurrence remains limited. **Aims**: We aimed to evaluate the association between CPAP adherence and risk of recurrent paroxysmal AF, and to compare time to first recurrence between patients with mean nightly CPAP use ≥4 h/night versus <4 h/night. **Materials and Methods**: In this prospective observational cohort (2017–2024), consecutive hospitalized and outpatient adults with severe obstructive sleep apnea (OSA; apnea–hypopnea index > 30 events/h) and documented paroxysmal atrial fibrillation (AF) were enrolled. Persistent and long-standing persistent AF were excluded to ensure a homogeneous population with respect to atrial substrate. OSA was assessed using home sleep apnea testing (ResMed ApneaLink), and all patients initiated continuous positive airway pressure (CPAP) therapy (ResMed AirSense 10). Objective adherence data were obtained via the ResMed AirView telemonitoring platform. Exclusion criteria included permanent AF, prior pulmonary vein isolation, central sleep apnea, left ventricular ejection fraction < 50%, end-stage chronic kidney disease (eGFR < 15 mL/min/1.73 m^2^ or dialysis), or inability to initiate or maintain CPAP therapy. Patients were followed for 12 months. The primary endpoint was time to first documented recurrence of paroxysmal AF (≥30 s on 12-lead electrocardiography or 24-h Holter monitoring). Progression to permanent AF, defined after unsuccessful rhythm control attempts and subsequent transition to a rate control strategy, was assessed as a secondary endpoint. Time-to-event analyses used Kaplan–Meier estimates with log-rank testing, and Cox proportional hazards regression adjusted for age, body mass index, apnea–hypopnea index, heart failure, left atrial volume index, and antiarrhythmic drug therapy. **Results**: The final analysis included 91 patients (mean age 62.15 ± 8.29 years; 68.13% men). Mean nightly CPAP use was ≥4 h/night in 49 patients and <4 h/night in 42 patients. During follow-up, paroxysmal AF recurrence occurred in 12/49 (24.5%) patients in the ≥4 h/night group and 16/42 (38.1%) in the <4 h/night group. Mean arrhythmia-free survival at 12 months was numerically higher in the ≥4 h/night group (11.25 vs. 10.51 months), without a statistically significant difference in Kaplan–Meier curves (log-rank *p* = 0.11). In multivariable Cox regression, binary adherence (≥4 h/night) was not independently associated with recurrence (HR 0.52, *p* = 0.13), whereas mean nightly CPAP use analyzed as a continuous variable remained independently associated with delayed recurrence (per 1-h increase: HR 0.66, 95% CI 0.48–0.91, *p* = 0.01). Progression to permanent AF occurred in 4/49 (10.0%) versus 9/42 (17.6%) patients, respectively (*p* = 0.29). **Conclusions**: In this real-world cohort of patients with severe OSA and paroxysmal AF, higher objectively measured CPAP exposure was independently associated with delayed AF recurrence when analyzed as a continuous variable, suggesting a graded association between objectively measured CPAP exposure and AF recurrence. Larger studies with extended follow-up and continuous rhythm monitoring are warranted to confirm long-term rhythm benefits and effects on AF progression.

## 1. Introduction

OSA is a highly prevalent sleep-related breathing disorder characterized by recurrent upper airway obstruction during sleep, resulting in intermittent hypoxia, sleep fragmentation, and autonomic imbalance [1]. Beyond its respiratory manifestations, OSA has important cardiovascular consequences and is now recognized as a major modifiable risk factor for AF, the most common sustained cardiac arrhythmia worldwide [2]. Epidemiological studies demonstrate a high prevalence of OSA among patients with AF, frequently exceeding 50%, underscoring a strong and clinically relevant association between these conditions [3]. The link between OSA and AF is supported by well-established pathophysiological mechanisms. Intermittent hypoxemia, exaggerated negative intrathoracic pressure swings, sympathetic activation, oxidative stress, and systemic inflammation promote atrial electrical instability and progressive structural remodeling, including atrial dilatation, fibrosis, and conduction heterogeneity, thereby facilitating both AF initiation and maintenance [2,4]. Moreover, the severity of sleep-disordered breathing and nocturnal hypoxemia appears to influence AF risk, suggesting a dose-dependent relationship between OSA burden and arrhythmogenesis [5]. Clinical evidence consistently indicates that OSA adversely affects rhythm control outcomes in AF. Untreated OSA has been associated with higher rates of AF recurrence following electrical cardioversion and catheter ablation compared with patients receiving CPAP therapy or those without OSA [6]. Observational studies and meta-analyses further suggest that CPAP therapy is associated with reduced AF recurrence and a lower likelihood of progression to more sustained AF forms, supporting the concept that effective treatment of sleep-disordered breathing may favorably modify the arrhythmogenic substrate [2,7]. These observations have led to the incorporation of OSA management into contemporary AF care guidelines [8]. However, evidence from randomized controlled trials (RCTs) has been less consistent, highlighting an important discrepancy between observational and randomized data. Short-term RCTs using continuous rhythm monitoring failed to demonstrate a significant reduction in AF burden with CPAP therapy despite effective OSA treatment [9]. Similarly, in patients undergoing pulmonary vein isolation, CPAP did not reduce early AF recurrence within the first post-ablation year [10]. These findings raise important questions regarding optimal endpoints, follow-up duration, and patient selection needed to adequately capture the potential benefits of CPAP on AF outcomes. Emerging data suggest that CPAP adherence and treatment duration are critical determinants of clinical benefit. Long-term adherence has been associated with a significant reduction in very late AF recurrence beyond the first post-ablation year, indicating that sustained therapy may be required to favorably influence atrial remodeling and long-term rhythm stability [11]. These findings highlight the complexity of CPAP-related effects on AF and suggest that recurrence-based endpoints may better capture the potential benefits of CPAP on AF outcomes. Collectively, these trial results may reflect endpoint selection (burden vs. recurrence), limited follow-up to capture remodeling, suboptimal adherence, and clinical heterogeneity, rather than definitive absence of a CPAP-related rhythm signal. Nevertheless, most available studies rely on binary adherence thresholds and provide limited real-world data on the relationship between objective CPAP exposure and clinically meaningful AF outcomes, particularly in patients with severe OSA and paroxysmal AF. This phenotype is clinically relevant because severe OSA is associated with pronounced intermittent hypoxemia and autonomic activation, while paroxysmal AF represents a stage at which upstream risk factor modification may still influence substrate evolution [12]. While prior observational and post-ablation studies have demonstrated associations between CPAP adherence and atrial fibrillation outcomes [6,7,11], prospective real-world data incorporating objectively quantified CPAP exposure and time-to-event recurrence endpoints remain comparatively limited, particularly in this high-risk phenotype. Accordingly, a clear gap remains regarding the impact of CPAP adherence on time to first AF recurrence in real-world clinical practice. Addressing this gap may improve risk stratification and inform integrated rhythm control strategies in this high-risk population. We hypothesized that greater objective nightly CPAP exposure would be associated with delayed time to first recurrence of paroxysmal atrial fibrillation.

## 2. Materials and Methods

This prospective observational cohort study was conducted in hospitalized and outpatient settings at a tertiary care center between September 2017 and December 2024, under real-world clinical practice conditions. The study was prospectively registered at ClinicalTrials.gov (identifier: NCT07358429). The study protocol was approved by the Committee on Ethics in Medical Scientific Research at the Medical University of Sofia (approval number 52BK-1047). All participants provided written informed consent prior to enrollment, and the study was conducted in accordance with the Declaration of Helsinki.

### 2.1. Study Population

Consecutive adult patients (≥18 years) with severe OSA and documented paroxysmal AF were prospectively enrolled. Severe OSA was defined as an apnea–hypopnea index > 30 events per hour. Paroxysmal AF was defined in accordance with contemporary guideline criteria as AF episodes terminating spontaneously or with intervention within seven days and documented by twelve-lead electrocardiography or twenty-four-hour Holter monitoring.

To ensure a homogeneous study population with respect to atrial substrate and disease stage, patients with persistent or long-standing persistent AF were not eligible. Patients with permanent AF were excluded, as were those with prior pulmonary vein isolation, central sleep apnea, reduced left ventricular systolic function (left ventricular ejection fraction < 50%), end-stage chronic kidney disease (estimated glomerular filtration rate < 15 mL/min/1.73 m^2^ or dialysis), or inability to initiate or maintain continuous positive airway pressure therapy.

### 2.2. Sleep Apnea Assessment and Cpap Therapy

Sleep-disordered breathing was assessed using home sleep apnea testing with ApneaLink™ (ResMed, Bella Vista, Australia), a validated portable respiratory polygraphy device recording nasal airflow, respiratory effort, heart rate, and peripheral oxygen saturation. ApneaLink™ has demonstrated high diagnostic agreement with in-laboratory polysomnography and is widely used for clinical screening and severity stratification of obstructive sleep apnea [13,14]. All recordings were reviewed for signal quality and automatically analyzed to derive the apnea-hypopnea index, oxygen desaturation index (ODI), and nocturnal oxygen saturation parameters. Following diagnosis, all patients initiated continuous positive airway pressure therapy using AirSense™ devices (ResMed, Australia) according to standard clinical practice. Objective adherence data, including mean nightly usage and the percentage of nights with therapy duration of at least four hours, were collected via the ResMed AirView™ cloud-based telemonitoring platform. Telemonitoring-based adherence assessment has been shown to provide reliable and clinically actionable data in real-world settings [15].

### 2.3. Follow-Up and Outcomes

Patients were followed for twelve months after initiation of continuous positive airway pressure therapy. Recurrence of paroxysmal atrial fibrillation was assessed during scheduled outpatient visits or symptom-driven evaluations and confirmed by 12-lead electrocardiography or 24-h Holter monitoring. AF recurrence was defined as an episode lasting ≥30 s documented on ECG or Holter recording. Continuous rhythm monitoring was not systematically performed.

The primary outcome was time to first documented recurrence of paroxysmal atrial fibrillation during follow-up. The secondary outcome was progression from paroxysmal to permanent atrial fibrillation within twelve months. Progression to permanent atrial fibrillation was defined as the transition from paroxysmal AF to a sustained AF state in which at least one documented attempt at rhythm restoration (pharmacological cardioversion, electrical cardioversion, or both) was performed and deemed unsuccessful, followed by a joint clinical decision by the treating physician and patient to abandon further rhythm control strategies and pursue rate control.

### 2.4. Statistical Analysis

Categorical variables are presented as counts and percentages, and continuous variables as mean ± standard deviation or median with interquartile range, as appropriate. Normality of continuous variables was assessed using the Kolmogorov-Smirnov test. Baseline comparisons between adherence groups were performed using the independent-samples t-test or Mann-Whitney U test for continuous variables and the chi-square test for categorical variables. Time-to-event analyses were conducted using Kaplan-Meier survival estimates and compared using the log-rank test. Cox proportional hazards regression models were applied to assess independent predictors of atrial fibrillation recurrence. Covariates were selected a priori based on clinical relevance and established associations with AF recurrence, including age, body mass index, apnea–hypopnea index, heart failure, left atrial volume index, and antiarrhythmic drug therapy. Mean nightly CPAP use was entered as a continuous variable (hours per night). Proportional hazards assumptions were verified prior to model interpretation. Statistical analyses were performed using IBM SPSS statistics (version 29.0.2.0), and a two-sided *p*-value < 0.05 was considered statistically significant.

## 3. Results

### 3.1. Baseline Characteristics

Baseline demographic and clinical characteristics of the study population according to continuous positive airway pressure adherence are summarized in Table 1.

The study population comprised 91 patients, of whom 49 were classified as having mean nightly continuous positive airway pressure use of at least four hours and 42 as having use below four hours. The two adherence groups were comparable with respect to baseline demographic characteristics. Mean age did not differ significantly between groups, and the proportion of male patients was similar.

The prevalence of major cardiovascular and metabolic comorbidities was balanced between adherence groups. No significant differences were observed in the rates of heart failure, chronic kidney disease, diabetes mellitus, ischemic heart disease, dyslipidemia, or arterial hypertension. Similarly, baseline atrial fibrillation risk and cardiac structural parameters were comparable. The CHA_2_DS_2_-VASc score, left atrial volume index, and left ventricular ejection fraction showed no statistically significant differences between patients with higher and lower therapy adherence.

Baseline severity of obstructive sleep apnea was also similar between groups. Both the apnea–hypopnea index and the ODI demonstrated comparable values, indicating a similar burden of sleep-disordered breathing at study entry.

Pharmacological therapy at baseline did not differ significantly according to adherence status. The use of oral anticoagulation, including non-vitamin K antagonist oral anticoagulants and vitamin K antagonists, as well as beta-blockers, antiarrhythmic drugs, and statins, was comparable between groups.

### 3.2. Primary Outcome: Time to First Recurrence of Paroxysmal Atrial Fibrillation

During the twelve-month follow-up period, paroxysmal atrial fibrillation recurrence occurred in 12 of 49 patients (24.5%) in the ≥4 h/night CPAP group and in 16 of 42 patients (38.1%) in the <4 h/night group. The absolute risk difference was 13.6%, corresponding to an exploratory number needed to treat approximately 8 patients over twelve months.

Kaplan–Meier survival analysis demonstrated a higher mean arrhythmia-free survival time in the higher-adherence group. At twelve months, mean arrhythmia-free survival was 11.25 months among patients using continuous positive airway pressure for at least four hours per night, compared with 10.51 months among patients with mean nightly use below four hours. Estimated 12-month recurrence-free survival was 75.5% in the ≥4 h/night group and 61.9% in the <4 h/night group. Median time to first recurrence was not reached in either group within the follow-up period.

**Figure 1:**
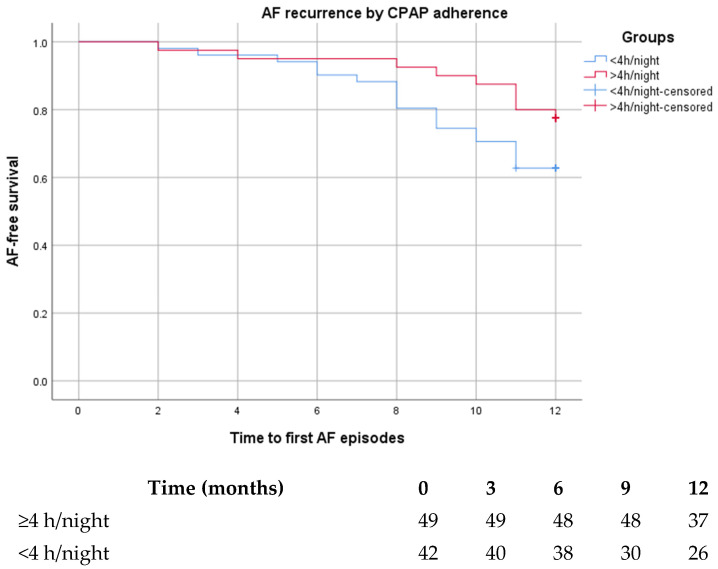
Kaplan–Meier curves for time to first recurrence of paroxysmal atrial fibrillation according to CPAP adherence (≥4 h/night vs. <4 h/night). Numbers at risk at 0, 3, 6, 9, and 12 months are shown below the curves.

Comparison of the survival curves using the log-rank test did not demonstrate a statistically significant difference in cumulative recurrence rates between adherence groups (log-rank *p* = 0.11). In unadjusted Cox regression, binary adherence showed a hazard ratio consistent with a protective direction, although not reaching statistical significance. The number of patients at risk at predefined time points is displayed below the survival curves. Although a gradual visual divergence of the curves appeared over time, the log-rank test did not demonstrate statistical significance, and therefore this finding should be interpreted cautiously (Figure 1).

### 3.3. Predictors of Atrial Fibrillation Recurrence

Results of the Cox proportional hazards regression analyses for predictors of time to first recurrence of paroxysmal atrial fibrillation are presented in Table 2.

In univariable Cox regression analyses, higher CPAP exposure was associated with a lower risk of atrial fibrillation recurrence. Mean nightly CPAP use, analyzed as a continuous variable, was significantly associated with recurrence risk, whereas binary CPAP adherence (≥4 h/night vs. <4 h/night) showed a consistent protective direction without reaching statistical significance. Age, body mass index, apnea–hypopnea index, heart failure, left atrial volume index, and antiarrhythmic drug therapy were not significantly associated with recurrence in univariable analyses.

In the multivariable Cox proportional hazards model adjusted for age, body mass index, apnea–hypopnea index, heart failure, left atrial volume index, and antiarrhythmic drug therapy, binary CPAP adherence was not independently associated with time to first atrial fibrillation recurrence (hazard ratio 0.52, *p* = 0.13). In contrast, mean nightly CPAP use remained independently associated with recurrence. Each one-hour increase in mean nightly CPAP use was associated with a 34% relative reduction in the risk of first atrial fibrillation recurrence (hazard ratio 0.66, 95% CI 0.48–0.91, *p* = 0.01).

### 3.4. Secondary Outcome: Progression to Permanent Atrial Fibrillation

Specifically, progression to permanent atrial fibrillation was documented in 4 of 49 patients (10.0%) with mean nightly CPAP use ≥ 4 h, compared with 9 of 42 patients (17.6%) with mean nightly use < 4 h. This difference did not reach statistical significance (*p* = 0.29).

## 4. Discussion

The present study sought to examine whether objectively measured CPAP exposure is associated with delayed recurrence of paroxysmal atrial fibrillation in patients with severe obstructive sleep apnea, whether continuous exposure metrics provide incremental information beyond dichotomized adherence thresholds, and whether this association persists after accounting for structural and rhythm-related variables.

Baseline demographic, clinical, and echocardiographic characteristics were well balanced between adherence groups. The predominance of cardiometabolic comorbidities and atrial structural remodeling in both groups is consistent with previously described AF populations with concomitant sleep-disordered breathing [16,17]. Obstructive sleep apnea and atrial fibrillation share upstream risk factors including obesity, hypertension, metabolic dysfunction, systemic inflammation, oxidative stress, and autonomic imbalance [1,4,12], mechanisms that are recognized contributors to atrial remodeling and arrhythmia susceptibility. This shared pathophysiological background is aligned with contemporary guideline-based approaches emphasizing risk factor modification in AF management [8].

The observed association between greater CPAP exposure and delayed time to first AF recurrence is directionally consistent with prior observational reports suggesting that treatment of sleep-disordered breathing may be linked to improved rhythm outcomes [6,7]. In contrast to earlier retrospective analyses, the present study prospectively evaluated a severe OSA cohort using telemonitoring-derived exposure metrics, which may provide a more granular characterization of nightly therapy use. Rather than establishing a causal effect, these findings are best interpreted as prospective real-world evidence that supports the hypothesis of an exposure–response relationship [18,19,20].

An important observation was the differential behavior of binary and continuous adherence metrics. While the ≥4 h/night threshold showed a protective direction without statistical significance, continuous nightly CPAP exposure remained independently associated with recurrence after multivariable adjustment. This pattern suggests that exposure–response relationships in OSA-related atrial remodeling may be graded rather than threshold-based. Similar challenges in detecting categorical treatment effects have been described in extended time-to-event analyses, in which separation of survival curves emerges over longer follow-up durations [21,22,23]. These data are consistent with the possibility that cumulative exposure, rather than dichotomized adherence definitions, may better reflect biological modulation of atrial substrate [22,23,24].

The association between CPAP exposure and recurrence remained after adjustment for left atrial size and antiarrhythmic drug therapy. Left atrial enlargement is widely regarded as a surrogate of structural remodeling and atrial cardiomyopathy [25], and rhythm control pharmacotherapy directly influences recurrence risk [20]. The persistence of the association after accounting for these factors suggests that the observed relationship is unlikely to be explained solely by baseline substrate burden or pharmacologic rhythm control.

From a mechanistic perspective, obstructive sleep apnea has been described as a contributor to atrial cardiomyopathy through intertwined pathways involving oxidative stress, endothelial dysfunction, inflammatory activation, and autonomic dysregulation [1,4,12,25]. Intermittent hypoxia may promote reactive oxygen species generation and impair nitric oxide bioavailability, contributing to endothelial dysfunction and sympathetic activation. These processes have been associated with microvascular dysfunction and regional perfusion mismatch, which may in turn facilitate atrial ischemia, fibrosis, and progressive electrical heterogeneity. Within this integrated substrate framework, sustained CPAP exposure may attenuate upstream drivers of remodeling; however, this mechanistic link remains inferential and should be confirmed in studies incorporating dedicated imaging and physiological endpoints.

The discrepancy between observational findings and randomized controlled trials evaluating CPAP in AF populations merits careful interpretation [9,10]. Randomized cohorts frequently include heterogeneous OSA severity, relatively short follow-up, and variable adherence, factors that may dilute biological effects. In addition, several trials have focused on arrhythmia burden rather than time to recurrence, endpoints that may capture different aspects of disease progression. Structural remodeling underlying atrial cardiomyopathy likely evolves over extended periods, and short-term analyses may underestimate delayed associations. Long-term adherence analyses in selected severe OSA populations have suggested potential benefit [11], supporting the hypothesis that exposure duration and intensity are relevant variables.

Within the broader rhythm control landscape, pulmonary vein isolation addresses dominant electrical triggers but does not directly reverse the underlying substrate. Larger left atrial volume and periatrial or epicardial adipose tissue characteristics have been associated with recurrence following ablation [26,27,28], underscoring the importance of structural and inflammatory remodeling. In this context, management of sleep-disordered breathing may represent one component of a comprehensive strategy targeting modifiable contributors to atrial substrate progression.

The numerically lower progression to permanent AF observed among patients with higher CPAP adherence is directionally consistent with upstream substrate stabilization, although this analysis was underpowered and should be interpreted cautiously. Contemporary longitudinal syntheses indicate that modest early rhythm differences may become more apparent with extended follow-up [28]. Overall, the present findings suggest an association between sustained CPAP exposure and arrhythmia-free survival in patients with severe OSA and paroxysmal AF, while highlighting the need for larger prospective cohorts and mechanistic investigations to clarify causality.

## 5. Limitations

This study has several limitations. Its observational design precludes causal inference and may be subject to residual confounding. CPAP adherence represents a non-random exposure and may partly reflect broader health engagement behaviors rather than an isolated therapeutic effect. Patients demonstrating higher adherence could also exhibit greater medication compliance, more proactive rhythm control management, or more consistent follow-up care, factors that were not fully captured in the present analysis despite balanced baseline characteristics and multivariable adjustment. The modest sample size, limited number of recurrence events, and twelve-month follow-up may have reduced statistical power, particularly for categorical adherence analyses, and may not fully capture longer-term structural remodeling processes or very late recurrence patterns.

Atrial fibrillation recurrence was assessed using intermittent ECG and 24-h Holter monitoring rather than continuous rhythm monitoring. AF episodes were considered recurrent if lasting ≥30 s and documented on ECG or Holter. Asymptomatic or undocumented episodes were not systematically captured, which may have led to underestimation of true recurrence rates and overall arrhythmia burden. However, rhythm assessment was performed uniformly across adherence groups within routine clinical practice, reducing the likelihood of differential detection bias. Therefore, while time to first documented recurrence may not fully reflect cumulative AF burden, the relative comparison between adherence groups remains internally consistent.

Although predefined clinical criteria were applied, the classification of permanent AF inherently involves a therapeutic decision and may be influenced by patient preference, comorbidity profile, and clinical judgment.

Residual apnea–hypopnea index and mask leak parameters were not systematically included in the statistical analysis; although mask fit was routinely optimized in clinical practice, the absence of these physiological metrics should be acknowledged as a minor limitation.

In addition, the single-center design may limit generalizability to broader and more heterogeneous populations.

Key strengths include prospective enrollment, objective telemonitoring-based assessment of CPAP adherence, use of time-to-event analyses, and focus on clinically relevant outcomes such as time to first recurrence and progression to permanent atrial fibrillation. Together, these features support the clinical relevance of the findings and provide a rationale for larger studies with extended follow-up.

## 6. Conclusions

In this real-world cohort of patients with severe obstructive sleep apnea and paroxysmal atrial fibrillation, greater objectively measured CPAP exposure was associated with delayed arrhythmia recurrence, suggesting a potential graded exposure–response association. Although binary adherence thresholds did not reach statistical significance, objectively measured nightly CPAP use remained independently associated with longer arrhythmia-free survival. These findings support the hypothesis that sustained CPAP exposure may be associated with improved rhythm stability; however, this association should be confirmed in larger prospective cohorts with extended follow-up to better define its potential role within integrated atrial fibrillation management strategies.

## Figures and Tables

**Table 1:** Baseline characteristics by CPAP adherence group (≥4 h/night vs. <4 h/night).

Variable	Overall (n = 91)	≥4 h/Night (n = 49)	<4 h/Night (n = 42)	*p*-Value
Age, years	62.15 ± 8.29	62.06 ± 8.22	62.26 ± 8.47	0.952
Male sex, n (%)	62 (68.13)	35 (71.43)	27 (64.29)	0.466
**Comorbidities**				
Heart failure, n (%)	33 (36.26)	18 (36.73)	15 (35.71)	0.920
Chronic kidney disease, n (%)	22 (24.18)	12 (24.49)	10 (23.81)	0.940
Diabetes mellitus, n (%)	35 (38.46)	18 (36.73)	17 (40.48)	0.715
Ischemic heart disease, n (%)	24 (26.37)	13 (26.53)	11 (26.19)	0.971
Dyslipidemia, n (%)	42 (46.15)	25 (51.02)	17 (40.48)	0.314
Arterial hypertension, n (%)	61 (67.03)	34 (69.39)	27 (64.29)	0.606
**Atrial fibrillation risk and cardiac structure**				
CHA2DS2-VASc score	2.74 ± 0.85	2.76 ± 0.85	2.71 ± 0.86	0.901
Left atrial volume index, mL/m^2^	37.06 ± 6.62	37.34 ± 6.73	36.73 ± 6.54	0.664
Left ventricular ejection fraction, %	57.77 ± 7.95	57.98 ± 7.07	57.52 ± 8.94	0.852
**Sleep apnea severity**				
Apnea–hypopnea index, events/h	64.01 ± 22.15	65.90 ± 23.09	61.80 ± 21.06	0.378
Oxygen desaturation index, events/h	57.13 ± 18.99	58.79 ± 19.72	55.20 ± 18.13	0.368
**Baseline pharmacological therapy**				
Oral anticoagulation, n (%)	60 (65.93)	30 (61.22)	30 (71.43)	0.306
NOAC, n (%)	52 (57.14)	28 (57.14)	24 (57.14)	1.000
Vitamin K antagonist, n (%)	10 (10.99)	3 (6.12)	7 (16.67)	0.178
Beta-blocker, n (%)	59 (64.84)	31 (63.27)	28 (66.67)	0.735
Antiarrhythmic drug, n (%)	54 (59.34)	26 (53.06)	28 (66.67)	0.188
Statin, n (%)	38 (41.76)	20 (40.82)	18 (42.86)	0.844
**CPAP therapy**				
Mean nightly CPAP use, h/night	4.16 ± 1.27	5.13 ± 0.75	3.03 ± 0.67	<0.001

CHA2DS2-VASc, Congestive heart failure, Hypertension, Age ≥ 75 years, Diabetes mellitus, Stroke/transient ischemic attack, Vascular disease, Age 65–74 years, Sex category (female); CPAP—continuous positive airway pressure; NOAC—non-vitamin K antagonist oral anticoagulant.

**Table 2:** Cox proportional hazards regression analysis for predictors of paroxysmal atrial fibrillation recurrence.

Variable	Univariable HR (95% CI)	*p*-Value	Multivariable HR (95% CI)	*p*-Value
CPAP adherence ≥ 4 h/night	0.58 (0.29–1.17)	0.12	0.52 (0.23–1.17)	0.13
Mean nightly CPAP use (per 1 h)	0.71 (0.55–0.92)	0.01	0.66 (0.48–0.91)	0.01
Age (per year)	1.02 (0.99–1.06)	0.21	1.04 (0.99–1.09)	0.14
BMI (per kg/m^2^)	1.03 (0.98–1.09)	0.18	0.97 (0.88–1.07)	0.57
AHI (per event/h)	1.01 (0.99–1.03)	0.22	1.01 (0.99–1.03)	0.26
Heart failure	1.29 (0.87–1.92)	0.19	0.85 (0.36–2.04)	0.72
Left atrial volume index	0.98 (0.94–1.02)	0.38	0.97 (0.90–1.04)	0.34
Antiarrhythmic drug therapy	1.03 (0.46–2.29)	0.94	0.84 (0.37–1.93)	0.68

HR—hazard ratio; CPAP—continuous positive airway pressure; BMI—body mass index; AHI—apnea–hypopnea index.

## Data Availability

Data cannot be shared for ethical/privacy reasons. The data underlying this article cannot be shared publicly due to ethical reasons. The data contain sensitive information and are associated with questionnaires completed by patients. The data will be shared upon reasonable request to the corresponding author, after any sensitive information has been removed.

## Abbreviations

The following abbreviations are used in this manuscript:

AF	Atrial fibrillation
AHI	Apnea–hypopnea index
BMI	Body mass index
CPAP	Continuous positive airway pressure
ECG	Electrocardiography
eGFR	Estimated glomerular filtration rate
ESC	European Society of Cardiology
HR	Hazard ratio
ODI	Oxygen desaturation index
OSA	Obstructive sleep apnea
RCT	Randomized controlled trial
